# Proposed management model for the use of telemonitoring of adherence to positive airway pressure equipment - position paper of the Brazilian Association of Sleep Medicine - ABMS

**DOI:** 10.5935/1984-0063.20200086

**Published:** 2021

**Authors:** Tatiana de Aguiar Vidigal, Evelyn Lucien Brasil, Morgana Nicolodelli Ferreira, Luciane Luna Mello-Fujita, Gustavo Antonio Moreira, Luciano F. Drager, Leticia Azevedo Soster, Pedro Rodrigues Genta, Dalva Poyares, Fernanda Louise Martinho Haddad

**Affiliations:** 1 Universidade Federal de São Paulo (UNIFESP), Departamento de Psicobiologia - São Paulo - São Paulo - Brazil.; 2 Universidade Federal de São Paulo (Unifesp), Dep. de Otorrinolaringologia e Cirurgia de Cabeça e Pescoço - São Paulo - São Paulo - Brazil.; 3 Hospital Israelita Albert Einstein, Dep. de pacientes graves e Terapia PAP -São Paulo - São Paulo - Brazil.; 4 Hipnos Assessoria e Produtos para o Sono, Adaptação e monitoramento Terapia PAP - Curitiba - Paraná - Brazil.; 5 Universidade Federal de São Paulo (UNIFESP) Departamento de Pediatria -São Paulo - São Paulo - Brazil.; 6 Sleep Laboratory, Pulmonary Division, Heart Institute (InCor), Hospital das Clinicas HCFMUSP, Universidade de São Paulo - São Paulo - São Paulo - Brazil.

**Keywords:** Obstructive Sleep Apnea, CPAP Adherence, Telemonitoring

## Abstract

This document “Proposed management model for the use of telemonitoring to positive airway pressure adherence” was prepared by a special commission of the Brazilian Association of Sleep Medicine, with the objective of recommending a follow-up model for patients undergoing positive airway pressure therapy using telemonitoring. This proposal was prepared based on a survey and analysis of the most up-to-date national and international literature and uses the best available evidence to facilitate the standardization of care by Sleep Science specialists with potential benefit for patients. Among the conclusions of the document, it is emphasized that telemonitoring is an important tool that allows health professionals trained in sleep-disordered breathing to remotely monitor PAP therapy, allowing prompt and, when necessary, daily adjustments to be made in order to increase adherence to treatment. The authors also conclude that the privacy of the data received and shared during the provision of telemonitoring must be respected by the physician or health professional trained in sleep, with the authorization of the patient and/or person responsible, who should be made aware of the short-, medium- and long-term provision of the service.

## INTRODUCTION

### Prevalence and classification of OSA

Obstructive sleep apnea (OSA) is characterized by recurrent episodes of partial intermittent obstruction of the upper airway during sleep, associated with hypoxemia, hypercapnia and sleep fragmentation^1^. Snoring can be associated with OSA and indicates a decrease or narrowing of the upper airway during breathing. The presence of five or more obstructive respiratory events (apneas, hypopneas and arousals associated with respiratory effort [RERA]) per hour is considered abnormal in adults. OSA is recognized as an independent risk factor for cardiac, metabolic, and neurological morbidities^2^.

In an epidemiological study by Tufik et al. (2010)^3^ in the adult population of the city of São Paulo, a prevalence of 32.9% of obstructive sleep apnea syndrome was observed and diagnosed by means of polysomnography. However, depending on the criterion used to define the disease, the prevalence of OSA in adults aged 30 to 69 years varies worldwide between 5% and 49% in men, and 2% and 35% in women, considering an apnea-hypopnea index (AHI) greater than 15 events per hour of sleep^3^. In a recent global assessment, it was estimated that almost 1 billion people have some degree of OSA, with about half of this population having moderate to severe forms of OSA^4^.

OSA can be classified as mild, moderate or severe, according to the frequency of respiratory events observed in polysomnography, quantified using the apnea-hypopnea index (AHI), or the respiratory disturbance index (RDI) OSA severity is defined as mild for an AHI greater than or equal to 5, and less than 15 events/h; moderate for an AHI greater than or equal to 15 events/h, and less than 30 events/h; and severe for an AHI greater than or equal to 30 events/h^5^. Traditionally, sleep studies have been categorized as type I, type II, type III or type I V. Type II studies use the same monitoring sensors as full PSGs (type I) but are unattended, and thus can be performed outside of the sleep laboratory. Type III studies use devices that measure limited cardiopulmonary parameters; two respiratory variables (e.g., effort to breathe, airflow), oxygen saturation, and a cardiac variable (e.g., heart rate or electrocardiogram). Type IV studies utilize devices that measure only 1 or 2 parameters, typically oxygen saturation and heart rate, or in some cases, just air flow^6^.

A diagnosis of OSA is confirmed if the number of obstructive breathing events seen on polysomnography is greater than 15 events/h, or greater than 5/h with complaints of non-restorative sleep, excessive daytime sleepiness, fatigue or insomnia, choking or suffocating, or reports of snoring and/or respiratory pauses described by a roommate during the patient’s sleep; and/or a diagnosis of hypertension, mood disorder, cognitive impairment, coronary artery disease, stroke, congestive heart failure, atrial fibrillation, and type 2 diabetes mellitus^7^

### Clinical consequences

OSA can affect various organs and systems, and symptoms can include snoring, excessive daytime sleepiness, non-restorative sleep, irritability, depression, memory deficit, difficulty concentrating, and decreased alertness^8,9^ Other outcomes include increased errors, reduced attention, impaired work efficiency, decreased quality of life, and an increased risk of automobile and work accidents^10,11^. The mechanisms attributed to these outcomes are intermittent hypoxemia, oxidative stress, sleep fragmentation, and increased intrathoracic pressure^12^.

Excessive daytime sleepiness (EDS) is one of the most prevalent and recognized consequences of OSA and is not correlated with the severity of OSA^13^. EDS can reduce productivity at home and at work, and increase the risk of car and work accidents^13,14^. One study reported twice as many occupational accidents, and an increased incidence of work-related vehicle accidents^15,16^. Absenteeism in patients diagnosed with OSA is greater compared to patients without OSA. A study demonstrated that individuals with OSA have longer medical leave (more than 30 days) compared to individuals without OSA^16^.

OSA is associated with cognitive impairment, its main determinants are intermittent hypoxia and sleep fragmentation The cognitive domains that are compromised due to OSA are attention and vigilance, long-term visual and verbal memory, and executive function^17,18^.

OSA is independently associated with metabolic syndrome and insulin resistance. Insulin resistance correlates positively with the severity of OSA, as measured by the AHI, the oxyhemoglobin desaturation index and the arousal index. The long-term effect of CPAP therapy on patients with established diabetes is not yet known, but some studies show that it can lead to improved insulin sensitivity^5,19^.

Evidence demonstrates a causal relationship between OSA and the incidence of systemic and pulmonary arterial hypertension, coronary heart disease, arrhythmia, heart failure, and stroke. In severe cases of OSA, there is an increased risk of death from cardiovascular events^20,21,22,23^ Studies have shown that using continuous positive pressure therapy (CPAP) for more than four hours a night reduces systemic blood pressure, the risk of recurrent atrial fibrillation, and fatal and non-fatal cardiovascular events, and increases the survival of apneic patients with stroke^22,24,25,26^. It is important to highlight that, in the context of secondary prevention, that is, patients with previous cardiovascular and cerebrovascular diseases, the benefit of OSA treatment in preventing fatal and non-fatal events is uncertain^27^.

### Indication of positive pressure treatment

Positive airway pressure (PAP) therapy is mainly indicated for the treatment of moderate to severe OSA^28^. PAP adherence is variable, ranging from 29 to 83% in the literature^29,30^. Although considered a highly effective treatment, the success of PAP treatment and the maintenance of its benefits depend on regular, daily use, and throughout the sleep period the minimum recommendation for PAP use is four hours per night on 70% of the nights^31^.

Several factors can influence PAP adherence. Nasal obstruction, occurring in up to 40% of patients, is associated with worse adherence^32^. Low adherence can also be explained by other adverse effects related to the use of the equipment, such as mask intolerance, dry nose and mouth, fragmented sleep, equipment noise, skin irritation, positive pressure intolerance - including difficulty in exhaling and air leakage from the mask or mouth^33,34,35^.

Psychological factors should also be considered in PAP adherence. Depression or mood disorders are not considered predictors of poor treatment, however, it has been demonstrated that the perception of patients about symptoms after the use of PAP therapy and side effects differs between adults with and without depression. Patients with depression who improved with the use of PAP reported improvement in daytime symptoms, while those with more strike depressive conditions did not report improvement in daytime symptoms. Individuals with type D personality (negative affectivity and social inhibition) reported more severity and perception of the side effects of PAP and lower adherence in the objective measure when compared to those without type D personality^25^.

The longer the use of PAP, the greater the benefits in relation to the improvement of daytime sleepiness (measured by the Epworth sleepiness scale, or the multiple sleep latency test) and quality of life associated with OSA (functional outcomes of sleep – FOSQ)^31^ Cardiovascular and mortality outcomes have also been shown to be better with increases in the number of hours of PAP use^22,36,37^. Studies have shown that good initial adherence is a predictor of long-term adherence, with adherence in the first month predicting satisfactory use over a year^38,39^. Considering the importance of optimal initial adherence, several interventions have been aimed at improving adherence to PAP. However, there are few controlled studies on behavioral therapies, mainly considering viability and cost^40^.

The telemonitoring strategy has been shown to be effective in increasing adherence to PAP therapy when compared to usual care^41^. The benefits of telemonitoring are early adjustments to PAP device parameters with the potential for increased adherence, reduced time spent visiting the health professional, reduced absenteeism at work, solves problems with distance from urban centers and traffic jam, and reduction costs with face-to-face visits^42,43,45,46,47^.

### Objective of this statement

Given the benefits of PAP in respect of OSA, and the challenges involved in adhering to therapy, the objective of this document is to recommend a model for using telemonitoring with patients receiving positive airway pressure therapy. It is a suggestion based on the best available evidence that can facilitate physicians specializing in sleep medicine, and the multidisciplinary team, to standardize care, with a potential benefit for patients.

## USE OF TELEMONITORING AS AN ADHERENCE TOOL

### Distance monitoring and data transmission systems

**Figure 1:**
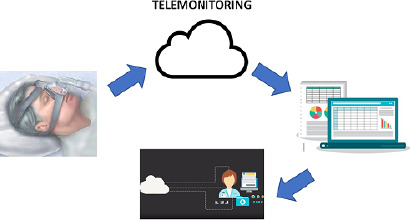
Telemonitoring system and transmission of analysis reports.

Telemonitoring is a pre-existing technology that manufacturers have added to their PAP equipment in order to promote adherence. It allows a range of information related to efficiency and adherence, as well as the adjustment of the user interface, the hours and days of use, and the therapeutic pressure (Figure 1).

Some manufacturers of PAP equipment use modems designed to transfer information to a cloud database via internet or Bluetooth connections. Other manufacturers have opted for transmission technology using GSM networks, which have the advantage of automatically transferring and receiving data.

These transmission systems make it possible to offer the service, without the provider or the companies that develop the technology incurring any costs, requiring only investment in monitoring instruments, such as computers, Tablets and cell phones with internet access and domestic Wi-Fi (broadband).

These systems may have some limitations in the service, due to failures of transmission of analysis reports and possible problems on reception. The different transmission systems have advantages and disadvantages listed in Table 1.

**Table 1:** Advantages and disadvantages of transmission systems.

Make	PAP PAP equipment model	Data transmission system	Advantages	Disadvantages
Fisher & Paykel	ICON	InfoUSB		Requires computer with a USB port; Use limited by technological resources; Transmission and Receipt of data require patient intervention; Failure of wired Ethernet cable, home Wi-Fi networks (broadband), and Bluetooth connection
Fisher & Paykel	SleepStyle	Bluetooth, Wi-Fi and 3G	Transfer (need for access to the App by the patient to release data) and data reception with need for patient intervention, using more data privacy.	Requires use of cell phone, use limited by availability of technological resources; Transmission and receipt of data requires intervention by patient; Failure of Ethernet cable, home Wi-Fi networks (broadband), and Bluetooth connection.
Philips Respironics	DreamStation	Bluetooth, Modem Wi-Fi and 3G	Flow curve analysis; transmission (need for access to the App by the patient to release data) and reception of data with the need for patient intervention providing more data privacy.	Requires use of cell phone, use limited by availability of technological resources; Transmission and receipt of data requires intervention by patient; Failure of Ethernet cable, home Wi-Fi networks (broadband), and Bluetooth connection.
ResMed	AirSense	GSM	Transmission and reception of data without the need for patient intervention	Power cut or loss of GSM network; Slow transmission and reception.

### Advantages of telemonitoring

Telemonitoring can improve access to health systems, reduce the time spent on medical visits and clinical investigations, and increase adherence to treatments in general It solves problems related to distances from urban centers, traffic congestion and missing work. The current position of the American Academy of Sleep Medicine (AASM) stresses the importance of telemonitoring in promoting a model of care in which specialized physicians and qualified sleep professionals can work together to improve the provision of healthcare for patients with sleep disorders in general. However, the most recent studies have focused on sleep-related breathing disorders^43,44^.

Telemonitoring of sleep-disordered breathing enables remote monitoring of PAP use, including assessment of data relating to adherence, leakage and therapeutic pressure levels .Prompt remote interventions allow adjustments to be made to the equipment parameters, with the potential to increase adherence, reducing in-person visit, improving treatment quality, and decreasing the rate of treatment drop-out^45,46,47^. In a study evaluating the impact of telemonitoring on adherence to CPAP with pressure above 16cmH_2_O, there was an increase in the use of CPAP of 114 minutes per night compared to individuals having face-to-face monitoring^48^.

To maintain increased adherence, it is suggested that telemonitoring should be continuous^49^. The implementation of telemonitoring of PAP data associated with distance education programs increased adherence in the first 90 days of treatment, and the likelihood of long-term use of the device (>360 days)^50^.

There is still no consensus in the literature regarding the benefit of using PAP therapy telemonitoring. A recent study found that in a population with OSA being treatment with PAP, there was no increase in adherence in the group randomized to telemonitoring, in comparison with those having face-to-face monitoring. In addition, telemonitoring was associated with lower patient satisfaction in relation to treatment^47^. In contrast, a recent meta-analysis compared studies using traditional monitoring with remote monitoring in patients with OSA Individuals who underwent telemonitoring showed greater adherence to PAP compared to those whose had traditional face-to-face monitoring meetings In the same study, it was shown that home titration with APAP was not inferior to supervised laboratory titration^51^. Titration with home APAP may be an option for titration in adults with moderate to severe OSA without significant comorbidities, such as congestive heart failure, chronic obstructive pulmonary disease, central apnea, hypoventilation, neuromuscular disease, and associated sleep disorders such as severe insomnia, parasomnias, and periodic leg movement. There was no clinically significant difference in adherence, sleepiness or quality of life^41,52^.

Two preliminary studies analyzed the profile of the Brazilian population and were consistent with the PAP adherence pattern according to the international literature. The findings of these studies support educational and motivational components for influencing compliance with CPAP and suggested the interdisciplinary approach for facilitate the identification of the difficulties faced by the patient^53,54^. More recently, a big data analysis included 4,181,490 patients from two developing countries (Brazil: 31,672; Mexico 16,934) and a developed one (US: 4,132,884) showed that the short- (90 days) and long-term (continuation therapy from day 91 to 1 year) CPAP adherence rates in Brazil and Mexico were similar to those achieved in the US^55^. Using US Center for Medicare and Medicaid Services (CMS) criteria (usage ≥4h/night on ≥70% of nights in the first 90 days), >80% of patients from all countries met CMS criteria at 3 months, with 1-year adherences rates of >75%. Interestingly, patients registered to use an engagement tool had a higher rate of CMS adherence and were twice as likely to achieve CMS adherence^55^.

Although the telemonitoring of CPAP is a recent innovation, and there is a shortage of scientific studies, the results so far point to a potential benefit in respect of adherence^55,56^ Moreover, the practicality of telemonitoring for the monitoring of patients with OSA is undeniable, especially in the initial period of adaptation, and can reduce travel and increase agility in the diagnosis of adverse events and the adjustment of settings.

### Limitations of the use of monitoring in clinical practice

The privacy of patient data is paramount. Information from the equipment is available to the patient, the company that supplies the PAP equipment, and to qualified healthcare professionals. Confidentiality is guaranteed by a one-time password. Any failures of this system must be identified and rectified by the partners involved. It is important to note that the data collected and transmitted by the manufacturers of the PAP equipment are encrypted, in line with the relevant legislation. One aspect to consider is the possible liability of health professionals for any lack of performance resulting from low adherence to PAP, and possible consequences related to health events or accidents associated with excessive sleepiness^47^. Such liabilities may eventually be addressed in the consent form suggested in the annex (Attachment 1)^44,57,58^.

Establishing wireless connections via Wi-Fi and/or Bluetooth on home networks requires the patient to use a cell phone application to enable access, which increases the complexity of its management Other systems require the patient to connect to the database via a computer, placing a memory device (pen drive) in a USB port for the information to be transmitted. Patients with limited resources, without a home computer or with a computer with an outdated operating system, may not be able to transmit data. The connection system via GSM makes the transmission of data easier, but it can be a problem in places with no connection to a cellular network. In addition, the equipment needs to be remain turned on after use to allow the transmission and reception of data.

### Inclusion of CBHPM code and remuneration of health insurers

As described above, the management of patients with sleep apnea by telemonitoring is a significant advance. In order to put in place a management and remuneration plan, the Brazilian Association of Sleep Medicine prepared a proposal for the management of patients with obstructive sleep apnea eligible for treatment with CPAP. The proposal was then ratified by the specialty societies (The Societies of Neurology, Neurophysiology, Otorhinolaryngology, Pulmonology, Clinical Medicine, Pediatrics and Psychiatry), and was presented to the National Commission of Medical Fees of the Brazilian Medical Association. There was a proposal to include a code in the Brazilian Hierarchical Classification of Medical Procedures (CBHPM), which details the remuneration for the management of patients with obstructive sleep apnea eligible for treatment with CPAP. In the minutes of the meeting of the technical chamber of the CBHPM of 25/02/2019, the procedure was approved and codified, with agreement on the classification and fees, becoming part of the CBHPM of 2019, according to normative resolution CNHM No. 039/2019 for doctors, hospitals and contracting entities (Attachment 2).

The code 2.01.02.15-1 was assigned to the management of patients with obstructive sleep apnea eligible for treatment with continuous positive airway pressure; associated with the inclusion of observation item, code 2.01.99.00-7, which refers to the frequency of the CPAP telemonitoring that is to be carried out weekly in the first month of treatment, subsequently every 3 months and then annually, not excluding the need to perform face-to-face consultations. The National Supplementary Health Agency (ANS) is responsible for incorporating health plan/ insurance operators’ procedures in the list. However, the use of the code must be negotiated with each insurer individually by the doctor, clinic or hospital. Its immediate use does not depend on ANS regulation, depending only on agreement between the operator and service provider.

## RECOMMENDED FOLLOW-UP

Telemonitoring allows the multidisciplinary team to participate more effectively in the therapy of patients using PAP Monitoring should be permanent, regardless of the specific period of time, as it is necessary to measure these results over the long term due to the chronic nature of OSA. A prompt and continuous approach to telemonitoring with frequent supervision in the first month is recommended, as this is the period that has been described as critical in relation to PAP adherence^38,39^. Frequent and detailed monitoring should continue with the objective of reducing treatment abandonment^59^. Remote assessment and reporting are recommended weekly in the first month, quarterly in the first year, and annually thereafter. It is recommended that presencial medical evaluations take place after 15 days of PAP use, and then at 30 days, 3 months, 6 months, 1 year, and are then maintained on an annual basis. The evaluation should be earlier if any irregularity in telemonitoring is observed.

The Tables below outlines the recommendations for follow-up through face-to-face evaluations and telemonitoring (Tables 2 and 3): NOTE: Telemonitoring is recommended to assess adherence and effectiveness on D60 (2^nd^ month), D120 (4^th^ month), D150 (5^th^ month), D210 (7^th^ month), D240 (8^th^ month), and D300 (10^th^ month) and, if necessary, earlier contact than at these suggested intervals if irregularities are observed, for example.

- Hours of inefficient use or non-use of PAP therapy;
- Fragmentation of sleep periods;
- Residual AHI >10/hour;
- Excessive leakage/escape.

**Table 2.** Face-to-face appointment.

Face-to-face appointment	Actions
D0	The PAP prescription, mask and pressure setting must be performed by a doctor. A health professional duly qualified in sleep- disordered breathing can provide services that include mask testing, PAP equipment adjustments and usage guidelines.
	Evaluation of clinical complaints and PAP parameters together with the prescribing physician
	Issue adherence and effectiveness report and detailed report.
	Evaluate:
D15 Annually	• Usage time: in the adherence and effectiveness report, analyze the average hours of use in the period, the percentage of days of use of >4h, and use of PAP for more than 4 hours per night in 70% of the nights evaluated. In the detailed report, note whether there is sleep fragmentation during the period of night use or in other periods, for example, disguised as good hours of use.
D30 (1st month)	• Residual AHI: considering the data from the polysomnography exam, check residual AHI (>10/h), weighing the indices for all events that can be detected, such as obstructive apnea, central apnea, hypopneas, RERA and Cheyne-Stokes respiration;
D90 (3rd month)	• Leakage/escape: in the reports, evaluate the total leakage value according to Table 4 of standardized maximum leakage, and check the efficiency of the mask adjustment and the residual AHI.
D180 (6th month)	Whenever necessary, perform adjustment of settings of the PAP equipment, according to the efficiency results presented in the reports generated by the equipment. This information must be registered in the medical record, and the patient must be aware of the adjustment made
D360 (12th month)	D30: Final adjustment of parameters of the PAP equipment, according to the efficiency results presented in the reports generated by the equipment.
Annually	Guidance regarding the replacement and cleaning of accessories, such as filters and mask.
	Completion of the medical report and send to patient.

**Table 3.** Evaluation by telemonitoring

Evaluation by telemonitoring	ACTIONS
	Telemonitoring to verify adherence and effectiveness of PAP equipment.
	Issue adherence and effectiveness reports, and the detailed report by a health professional duly qualified in sleep-disordered breathing.
	Evaluate:
D3 and D5*	• Usage time: in the adherence and effectiveness report, analyze the average hours of use in the period, the percentage of days of use of >4h, and use of PAP for more than 4 hours per night in 70% of the nights evaluated. In the detailed report, observe whether there is sleep fragmentation during the period of night use or in other periods, for example, disguised as good hours of use;
D7	• Residual AHI: considering the data from the polysomnography exam, check residual AHI (>10/h), weighing the indices for all events that can be detected, such as obstructive apnea, central apnea, hypopneas, RERA and Cheyne-Stokes respiration;
D21	• Leakage/escape: in the reports, evaluate the total leakage value according to Table 4 of standardized maximum leakage, and check the efficiency of the mask adjustment and the residual AHI.
D30 (1st month)#	Supervision and early contact if irregularities are observed:
D90 (3rd month)	• Hours of inefficient use or non-use of PAP therapy;
D180 (6th month) #	• Residual AHI>10;
D270 (9th month)	• Excessive leakage/escape.
D360 (12th month) #	Compare information with the previous evaluation period, if an adjustment is made.
	Remote adjustment of settings of the PAP equipment, according to the efficiency results presented in the generated reports, if necessary. Arrange face-to-face medical consultation if irregularities observed such as use for less than 70% of the total sleep time, persistent excessive leakage, the appearance of central respiratory events, or fragmentation of sleep periods.

* Strategic monitoring on days D3 and D5 to verify adherence and effectiveness, and early contact before D7 when irregularities are observed;

# On D30, D180 and D360, in addition to telemonitoring, arrange face-to-face consultation

## FLOWCHART: SEE BELOW A GUIDE FOR PAP THERAPY FOLLOW-UP.: STRATEGIES

Depending on the model of the equipment and the patient’s need, the doctor or qualified health professional has some resources that can be used to support the patient’s better adherence to the PAP equipment and therapeutic efficacy, such as: - mask fit, confort setting, humidifier, pressure change, improve nasal permeability, and medications.

### Mask fit

In the initial evaluation, a mask test is performed in order to try and determine the appropriate type, brand and size of mask considering the presence of any nasal symptoms and the size of the patient’s face. The preference is for the nasal model, rather than the oronasal model, due to its greater effectiveness, reduced chance of aerophagia, less leakage, and greater long-term adherence^60,61^.

The intranasal prong/cushion model is initially indicated at lower PAP pressures and according to nasal permeability, and can be used at higher pressures, but with less comfort in these cases.

Poor adjustment of the mask can lead to excessive leakage, leading to a reduction in efficacy and only partial resolution of obstructive events and, consequently, low adherence to therapy^62^ The patient and their partner should be instructed on the importance of the mask properly fitting the face. Nasal symptoms should always be reassessed, especially in the presence of excessive leakage. It may be necessary to change the type of mask if leakage persists Excessive leakage can also be due to wear to the mask’s silicone material, requiring replacement. If the leak is oral, a chinstrap can be used, but the results that are not always satisfactory^63^.

### Comfort setting

Pressure relief is a technology available in PAP devices to increase breathing comfort, and can involve expiratory relief, inspiratory and expiratory relief, decreased pressure during wakefulness and ramp. Studies suggest that these technologies do not substantially improve treatment adherence over fixed pressure PA P ^61,63^. However, some patients perceive greater comfort with expiratory relief, which may contribute to improving adherence to PAP therapy^64^.

### Use of a humidifier

The use of a humidifier can encourage greater adherence to PAP therapy, especially in patients with complaints of nasal and oral dryness^66^.

### Pressure change

The positive airway pressure therapy can be initiated using either APAP at home or in-laboratory PAP titration in adults with OSA and no significant comorbidities. Meta-analysis demonstrated no clinically significant differences in adherence, sleepiness, or QOL between APAP at home and in-laboratory PAP titration^51^. When APAP is implemented, the clinician is strongly encouraged to monitor the clinical response, PAP usage and therapy data within the first few weeks to make necessary PAP adjustments when indicated respecting of the minimum and maximum pressure adjustments^41^.

In the case of an AHI greater than 10/h, defined as the maximum tolerable AHI limit by the American Thoracic Society, in 2013^59^, telemonitoring becomes a useful tool to identify a pressure of subtherapeutic use, and allows pressure to be increased remotely to try to control respiratory events The therapeutic pressure setting must always be reevaluated if there is inadequate adherence and/or excessive leakage Pressure reduction, when possible, reduces leakage and can optimize adherence

### Nasal permeability

Nasal assessment is recommended whenever there is nasal obstruction and difficulty in using a nasal mask, discussing intervention with the prescribing physician if nasal symptoms persist^67^.

The question of whether upper airway permeabilization after nasal surgery can improve PAP adherence is a controversial one. However, surgery can increase the comfort of the use of PAP, and allow a reduction in therapeutic pressure levels, which could increase by 0,62h the duration of use of these devices^68^, especially in patients who need higher pressures^69,70^.

### Sleep inducing medication

The use of hypnotics during titration or in the first weeks of CPAP use can be helpful, increasing the hours of use of the device. However, it is not clear in which group of patients this strategy should be used, and should be evaluated by the physician responsible when necessary^71^. One study evaluated the use of sleep-inducing medication during split-night polysomnography, and despite the reduction in sleep latency, it did not increase pap adherence in the short term^72^. Bradshaw et al. (2006)^73^ concluded that the administration of an oral hypnotic agent did not improve initial CPAP compliance in men with OSA. Since there is no definition in the literature regarding the use of hypnotics to increase PAP compliance, in the presence of difficulty in initiating sleep, cognitive behavioral therapy may be useful^74^.

## REPORT

When analyzing the data that is stored in the cloud, the statistical data is visualized in a detailed report and, together with the clinical data, a personalized report is issued to identify the necessary adjustments to maintain effective treatment and allow remote assistance to the patient.

The medical report must be made and sent to the patient and to the health insurer weekly in the first month (Table 5), quarterly in the first year (Table 6), and thereafter it must be issued annually. It should contain the following information:

- Patient complaints from in person consultation,
- Report of the presence of snoring with the use of PAP and differentiated from leakage noise,
- An assessment of excessive daytime sleepiness,
- Strategies used such as pressure change, level of comfort relief, changes to ramp time, the use of a different mask, or specialized nasal evaluation,
- Guidance on care for equipment, mask, and accessories if necessary,
- Defined schedule for filter and mask changes
- Establish date of return in person or remotely.
- Rate PAP adherence:

**Table 5:** Report weekly in the first month.

	D7	D15	D21	D30
Days used				
Days not used				
Mask				
Pressure mode				
Percentile pressure 95				
Median pressure				
Average usage per night				
Median usage per night				
% nights of use of ≥4 hours				
Pressure relief				
Average leak				
AHI				
Obstructive apnea index				
Hypopnea index				
Central apnea index				

**Table 6:** Report quarterly in the first year.

	D90	D180	D270	D360
Days used				
Days not used				
Mask				
Pressure mode				
Percentile pressure 95				
Median pressure				
Average usage per night				
Median usage per night				
% nights of use ≥4 hours				
Pressure relief				
Average leak				
AHI				
Obstructive apnea index				
Hypopnea index				
Central apnea index				

Adherence: ( ) Good ( ) Poor

Leakage: ( ) Normal ( ) High

AHI: ( ) Normal ≤10/h ( ) High >10/h

## FINAL CONSIDERATIONS

Telemonitoring is an important tool that allows the physician or health professional properly trained in sleep disorders, to remotely monitor PAP therapy, allowing quick, timely, and daily adjustments when necessary in order to increase adherence to treatment. In addition, considering cost-benefit when compared to usual care, there is a reduction in the time spent visiting the doctor or health professional, reducing absenteeism at work, solving the distance from urban centers and congestion and reducing costs with face-to-face visits.

The privacy of the data received and shared during the telemonitoring process must be respected by the physician or health professional duly trained in sleep-disordered breathing, with the authorization of the patient and/or responsible person aware of the short, medium and long-term service provision.

Telemonitoring provides early interventions and permanent access to adherence and efficacy in any age group. When evaluating the use of this tool in adults and older adults, it is observed that the elderly adopt this modality frequently, but find greater technological limitations and the compliance rates may be lower with insomnia symptoms and cognitive impairment. There is no contraindications to remote monitoring.

It is important to emphasize that in the first 30 days, frequent observation, with detailed reviews of parameters such as leakage, residual AHI and hours of use, always correlated with clinical findings, help in the process of adherence and the efficiency of PAP therapy.

Recent evidence using de-identified telemonitoring data from more than 31,000 Brazilian patients with OSA showed higher than initially expected rates of good adherence rate to positive therapy at 90 days and 1-year^55^. Support programs that enable patients to track their CPAP therapy appear to be beneficial and should be further investigated and utilized^55^. Further studies are necessary to understand the role of each professional involved in the adherence process, how the resources presented by each manufacturer can be useful on a case-by-case basis, and the interaction of the multidisciplinary team. They should also evaluate the role of face-to-face and remote monitoring in adherence to PAP therapy.

## Figures and Tables

**Table 4:** Maximum leak threshold.

Equipment	Mask type	Leakage/escape
ResMed	Nasal/pillow	24 l/min
	Oronasal	36 l/min
PhilipsRespironics	Nasal/pillow	1 hour of a large leak or 60 l/min
	Oronasal	1 hour of a large leak or 60 l/min
Fisher & Paykel	Nasal/pillow	60 l/min(lcon)/leak up to 20% of time (SleepStyle)
	Oronasal	80 l/min(lcon)/leak up to 20% of time (SleepStyle)

**Figure 2:**
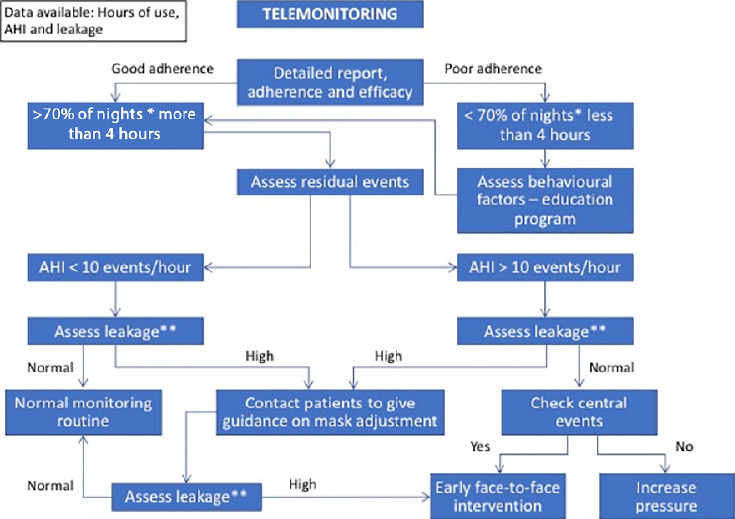
Flowchart - see above a guide for PAP therapy follow-up. * Every night of sleep. Reinforcing that use above 70% for of all nights during the entire period of sleep is recommended. ** Standardized excessive leak attachment.
